# A 16-Year Chronicle of Developing a Healthy Workplace Participatory Program for *Total Worker Health*^®^ in the Connecticut Department of Correction: The Health Improvement through Employee Control (HITEC) Program

**DOI:** 10.3390/ijerph21020142

**Published:** 2024-01-27

**Authors:** Martin Cherniack, Sara Namazi, Matthew Brennan, Robert Henning, Alicia Dugan, Mazen El Ghaziri

**Affiliations:** 1 Division of Occupational and Environmental Medicine, University of Connecticut Health Center, Farmington, CT 06030, USA; brennan@uchc.edu (M.B.); adugan@uchc.edu (A.D.); 2 Department of Health Science, College of Health & Wellness, Johnson & Wales University, Providence, RI 02903, USA; sara.namazi@jwu.edu; 3 Department of Psychological Sciences, University of Connecticut, Storrs, CT 06269, USA; robert.henning@uconn.edu; 4 Solomont School of Nursing, University of Massachusetts Lowell, Lowell, MA 01854, USA; mazen_elghaziri@uml.edu

**Keywords:** Participatory Action Research (PAR), Design Team (DT), Health Mentoring Program (HMP), Participatory Ergonomics, *Total Worker Health* (TWH), Healthy Workplace Participatory Program

## Abstract

Health Improvement Through Employee Control (HITEC) is a 16-year program directed toward the health of corrections personnel and developed through the application of the principles of Participatory Action Research (PAR) and participatory ergonomics. Its impetus has always been the adverse health status of the corrections workforce: early mortality, depression, obesity, and hypertension. The HITEC program trained small “Design Teams” (DTs) of front-line personnel in participatory methods for intervention design for health improvement and organizational change in line with the *Total Worker Health*^®^ principles. Periodic surveys and physical testing were introduced for longitudinal assessments. Comparative interventions at comparable sites included DTs without a priori assignation, problem-focused kaizen effectiveness teams (KETs), and bargaining unit-centered DTs. DT resilience and the replacement of members who transferred facilities or retired was aided by novel cooperative administrative structures. DT-generated interventions included stress lounges, changes in critical event report writing, a joint program with trained inmates to improve air quality, and training in staff mental health and sleep behavior. A specialized peer-to-peer Health Mentoring Program (HMP) paired new officers with trained peers. Many interventions and program features were institutionalized, thus improving prospects for self-supporting program longevity. Participatory interventions designed and supported by the corrections workforce were found to be both feasible and exceptionally effective.

## 1. Introduction

In October 2008, investigators from The University of Connecticut initiated a series of interventions with the Connecticut Department of Correction (CT DOC) to improve staff working conditions and health. Bearing the acronym of Health Improvement Through Employee Control (HITEC), the collaboration featured development, implementation, and evaluation of interventions that combined workplace health and safety with workforce well-being. The inception concept originated in a cooperative labor-management approach that integrated Participatory Ergonomics (PE) with the individualized focus of Health Promotion (HP) programs. Their combination was termed PExHP, or more descriptively as Occupational Safety and Health and Worksite Health Promotion. The HITEC concept was consistent with principles advanced by the National Institute for Occupational Safety and Health (NIOSH) *Total Worker Health* (TWH) program [1].

The first iteration of HITEC in 2006 was a comparison of two intervention approaches, each introduced into a separate corrections facility: (1) a best practices site intervention at Site A and (2) a participatory intervention at Site B. Site A introduced a set of combined environmental and individual well-being interventions that featured a management team working in collaboration with a correction officers (COs) bargaining unit. Site B interventions were chosen without prior screening by a participatory team of front-line level workers, termed a Design Team (DT), which was aided by study team facilitators. Because the best practices site was a distillation of successful interventions from the published literature, and our internal experience with weight loss interventions [2,3], we had anticipated that the results would be more favorable than those originating from the more laissez-faire DT approach. There were structural factors that suggested superior efficacy for the best practices site. Implementation was top–down. It began with a strong administrative endorsement, management “buy-in”, and utilization of established internal communications vehicles. There was also a relative stuffing with extramural resources, such as visits by health coaches and athletic trainers, that, by design, were not automatically available to the self-contained DT at Site B.

The first major intervention at the best practices Site A began with a voluntary survey and physiologic testing. A USD 50 incentive was offered. Testing was followed by an invitation to attend a private review of individual results with a nurse counsellor. The last step was to offer an appointment with a nurse health coach assigned to supervise a longer-term personal health program. In all, 161 of 340 (47%) eligible staff members elected to participate voluntarily in the survey and testing efforts. Fifty-two (52) participants chose to attend their individual session to review results. Only nine members of the workforce opted for health coaching. The low response rate qualified this administratively conceived top–down program as a failure. This unsatisfying response occurred despite the introduction of several consensus best practices for workplace health promotion: substantial investment of resources, provision of confidential health results with normalized comparison, and the opportunity for an individualized health maintenance plan. The experience seemed to confirm an observation made by other investigators that COs were diffident or fatalistic towards their own health [4,5]. Consequently, the administrative best practices approach was terminated.

Fifteen years later, in February 2023, HITEC, now middle-aged and seasoned, had passed through several phases of successes and failures, sequentially named HITEC 1–4. A DT of COs concluded three years, 2020–2023, of preparatory work with initiation of a peer support intervention, called the Facility Training Officer (FTO) program. The FTO was not entirely de novo, having been built within a framework of successful DT participatory work (see Table 1) and an experience with peer mentoring in HITEC 2 (see Table 1). The core idea was to train and place 3–4 COs as peer counselors in each corrections facility.

The practice threshold required extensive training and certification in peer counseling to effectively triage mental health problems, to assist work and family relations, and to offer financial and lifespan planning. The program was jointly sponsored by a union DT, the “Ambassadors”, and the CT DOC Training Academy (TA). The TA had expanded its conventional inception and certification programs into a quality-of-life program, advised and staffed by workforce peers. In essence, the FTO program was a unique bottom–up experiment previously untried in American corrections. The high level of DT sophistication and effective co-development with managerial groups were inconceivable and unrealizable at HITEC’s onset.

Another DT of unionized corrections supervisors entered 2023 with an annual series of integrated interventions for sleep hygiene, stress reduction, improved civility, and multiple internally designed surveys. The facilitation of work organizational change coupled with responses to individual needs fulfilled what had been attempted and had failed in 2008.

What happened between 2008 and 2023? Over 15 years, there had been continuous engagement between university investigators, corrections management, and labor representatives in the HITEC program. There had been experimentation with and an evaluation of different participatory intervention formats. Some participatory interventions had been abject failures. Others had succeeded in part but were incomplete or were not sustained. There were workforce-centric programs that were enthusiastically adopted but lacked measurable positive health effects. Research challenges included the lack of suitability of standardized survey measures in the corrections environment. There were also proposed interventions that reflected a high level of workforce creativity but had impractical resource expectations. In the end, however, a highly successful approach to the workforce control of health and well-being had emerged that was characterized by administrative commitment to needed resources and to flexible facilitation. Effectiveness required an ongoing commitment to cultural change and new organizational structures supporting organizational adaptability. What follows is a summary and interpretation of this 15-year developmental process. The impossible had proved to be possible. Our next goal is to identify patterns and formalize mechanisms to shorten the period for program adoption by other corrections systems as well as to ensure program sustainability.

PAR and PE have been used liberally in the manuscript, and some elaboration of the terms likely would be useful. There is no established definition of PE, but the practice of engaging the workforce in revising the physical workplace has an established tradition in labor management cooperation [6,7]. It belongs to the category of practices that the World Health Organization has termed “social health promotion” [8]. PE applications range from primarily technology-based problem solving to worker-centric evaluation and change [9,10]. We use the term as both a shorthand for workforce participation in work design and to increase familiarity with the concept of workforce participatory action. The practical application to HITEC is limited. In HITEC 2, the physical environment task was the sole specific application of PE.

The relevance of PAR to HITEC is not completely straightforward. When Kurt Lewin [11] introduced the concept of PAR after WWII, it was an attempt to integrate two desirable but potentially contradictory concepts: (1) research leading to practical outcomes associated with an identified social need; and (2) the direct engagement of the study population in study design and formulation. The implicit value placed on population engagement, situational specificity, and democratic determination of study aims potentially generates conflict over conditions for participation and adoption of research tools that appear redundant and may defy the common sense of the real world [12,13]. Other conflicts over setting priority include the blinding or maintenance of control populations and use of redundant and lengthy validated surveys [14,15]. An implementation of action research has worked particularly well in education, where the separation of an investigator from the action plan is implicit. Alternatively, participatory research, represented by Community-Based Participatory Research (CBPR), has as its goals improvement in research quality and inclusion, but it does not require the population to be defined as a priority [16]. A different set of considerations arose for HITEC when PAR principles were translated into the workplace. On the one hand, the necessary confinement and central experience of the workforce introduced a natural focus for constituency, physical locale, and finitude of problems. As other investigators have pointed out, training and learning processes are key parts of the work experience and establish intuitive acceptance of structured and gradualist approaches [17,18]. In the following summary of the HITEC program, the acceptance of DTs analyses and iterative proposals can be understood as expressions of worker-centricity.

Finally, this manuscript is a historical summary of the two-decades-long process. There are central themes, particularly the importance of and evolving structure of the integration and training of managers. It is not by any measure a recapitulation of results from our previous work, which is the reason that the results section is short and deals with non-overlapping points of presumed interests. Our body of work is represented in Table 1.

### Background: The HITEC Program

**Figure 1:**
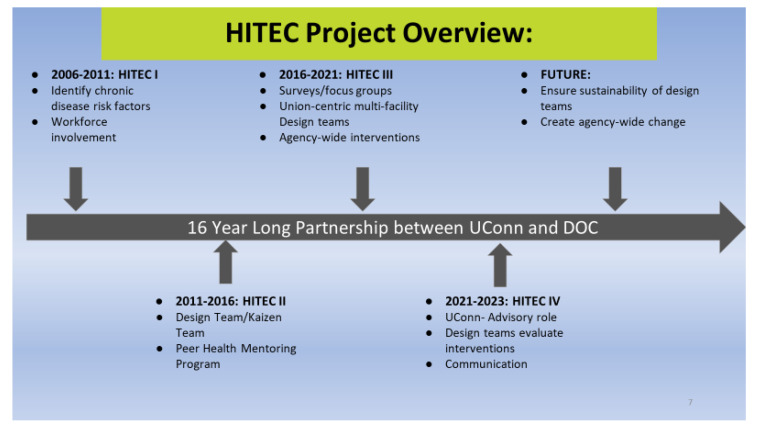
HITEC 2006–2023.

HITEC is an applied research-to-practice program initially developed from the Center for the Promotion of Health in the New England Workplace (CPH-NEW) (https://www.uml.edu/research/cph-newaccessed on 13 March 2023). Work on HITEC falls into four intervals: HITEC 1—2006–2011, HITEC 2—2012–2016, HITEC 3—2016–2021, and HITEC 4—2021–2023. The specific intervals are artificial, reflecting the duration of a discrete grant-funding period and requisite renaming. HITEC 1–3 (2006–2021) was funded originally by The National Institute for Occupational Safety and Health (NIOSH)’s *Total Worker Health* (TWH) program. The TWH program recognizes work as a social determinant of health and aims to protect the safety and health of workers and advance their well-being by integrating traditional workplace safety and health with individual health improvement [19]. HITEC 4, the current operational phase, is funded by the State of Connecticut under an agreement between the University of Connecticut and CT DOC. HITEC 1 and 2 were conducted at the two comparison sites. Facility-wide surveys were conducted with several hundred members of the workforce representing both sites, and at five points in time. Thus, in addition to the intervention comparisons, there was an 8-year longitudinal workforce assessment from 2008 to 2016. HITEC’s four iterations are captured in the following illustration (Figure 1).

**HITEC 1**. In its first iteration in 2006, HITEC began with a baseline health status survey at the two intervention sites. The sites were selected as an optimal pair from 18 possible corrections facilities in order to control for less confounded outcomes analyses, including facility size, security level, and age of facility and staffing. As noted, HITEC 1 entailed the comparison of an administratively conceived occupational safety and health and worksite health promotion/best practices professional program at one corrections facility (Site A) with an intervention program (Site B) directed by a CO-centered DT [1]. The DT was internally generated from the workforce through bargaining unit recommendation and open solicitation [20]. Site B adhered to principles of participatory action research (PAR), wherein the workforce developed interventions [21]. In HITEC 1, health outcomes were measured using surveys, focus groups, and interviews; physiologic performance testing; and a standardized Health Risk Appraisal (HRA). The baseline health results shocked staff and administrators. The sampled population of 326 COs and supervisors was in markedly poor health despite being relatively young—mean age, 40.0 years (SD = 7.2). Eighty-five percent (85%) were overweight or obese (BMI > 30); 35% were hypertensive, 56% were pre-hypertensive, and 31% reported symptoms consistent with clinical depression. In a separate analysis, comparing all CT DOC male COs with age-matched public sector peers, life expectancy was reduced by 12.6 years. In a coincident survey of a cohort of manufacturing workers, increases in blood pressure, in body mass, and in musculoskeletal symptoms were precocious in corrections personnel, with onset preceding the non-corrections group by 10–20 years (see Table 1). Baseline health status was identical at Sites A and B.

**Figure 2:**
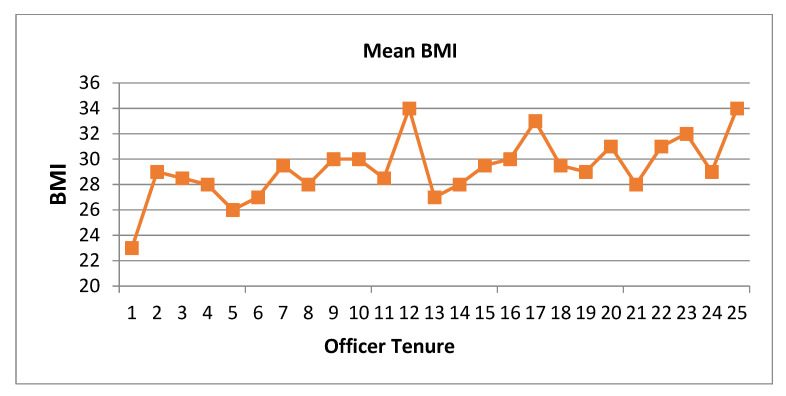
BMI and Employment Tenure.

In the 2008 baseline workforce survey, at both the best practices and participatory sites (Sites A and B), marked abnormalities already had occurred early in the work tenure, being detectable within the first 5 years of employment, as depicted for BMI in Figure 2.

HITEC 1 included three rounds of assessment through surveys and physiologic testing. Environmental sampling included indoor air quality (IAQ) and noise exposure measurement, both as area and some individual sampling. Variations of these baseline measurement modalities were revised and re-introduced in later applications of the HITEC program coincident with directed interventions. There were situational restrictions. Most individual environmental exposure assessment and measures of physical activity through data logging proved impossible at DOC facilities due to security prohibitions applied to worn devices. A description of assessment metrics follows under *Methods*.

HITEC 1 proceeded with two core principles: (1) program development through the central mechanism of workforce engagement and (2) the maintenance of a worker-centric orientation through issues identification and implementation. The two core principles were continued in later program phases. In the corrections milieu, this has meant that the health focus is on the workforce, exclusive of the inmate population, and that participatory initiatives for organizational change originated in DTs on a grass roots basis rather than as top–down administrative initiatives.

In **HITEC 2**, top–down administrative interventions were abandoned because there was a clear preference by both study populations at Sites A and B for the workforce-centric DT approach. The survey tools and group mechanisms employed in HITEC 2 evolved from HITEC 1, insuring the continuity of study metrics. There was a discontinuity between HITEC 1 and HITEC 2 that reflected the artificiality of the funding cycle, and also importantthe absence of a formalized study oversight group that assured the structured participation of DTs, union, management, and academic investigators.

Each intervention program followed a Participatory Action Research (PAR) approach. The participatory site (Site B) was represented by its ongoing DT consisting of COs only. At the comparison facility (Site B), there was a conversion to a series of joint labor management “kaizen effectiveness teams” (KETs) assembled around a pre-selected set of specific intervention themes determined to be high priorities in HITEC 1. Site B KETs were allocated a fixed 3-month time frame for planning an intervention (or interventions) to address each theme. Each KET was disbanded at the end of its fixed time frame. In contrast, at Site B, the participatory site projects were variably self-paced provided that a similar menu of interventions was completed by the end of the study period. Thus, the DT at Site B was not time-limited in problem solving duration for each area but was expected to complete the same four interventions by the project’s endpoint. A fuller description of the kaizen process used in HITEC appears in Table 1: Process evaluation of two participatory approaches: Implementing Total Worker Health interventions in a correctional workforce. The kaizen process used in HITEC was invariably longer than the flash interventions often practiced in industrial workplaces. Table 2 provides a comparison of the DT and KETs.

***DT and KET Comparison***. Inter-site comparability was maintained by assigning the same four specific intervention themes at both sites: (1) the physical environment, (2) weight loss and nutrition, (3) physical fitness, and (4) injury reduction secondary to violence.

***The Health Mentoring Program (HMP)***. During HITEC 2, the CO unions, influenced by evidence for adverse health effects early in employment, proposed a system-wide peer-to-peer mentoring intervention for new COs. HITEC 2 staff partnered with union leadership to vet and train established line-level staff for peer-to-peer cadet mentoring. The program, which was contained in the first year of new officer employment, included baseline, and then follow-up surveys and physical re-assessments at 1 and 5 years. The Health Mentoring Program (HMP) had a maximum and optimal duration of one year. Since the HMP was fully voluntary, a mentor–mentee full-year relationship was not obligatory. The HMP included quarterly field visits by research staff to survey and evaluate the mentor–mentee relationship and to update mentor performance for quality improvement. For purposes of comparison, non-mentored “control” groups received the same educational materials as mentees and relied on conventional on-the-job training (OJT). To clarify terminology, the comparison study design juxtaposed the mentored or Personalized Follow-Up (PFP) arm with the non-mentored control or Standardized Follow-up Arm (SFP). To prevent cross-contamination, mentored and non-mentored populations were entry class inclusive, so that mentored and non-mentored cadets did not overlap temporarily. The 5-year follow-up testing was consistent with the age-tenure disease induction period measured in the cross-sectional population study. The program concept and its use have been described in detail. (https://www.uml.edu/Research/CPH-NEW/Resources/Corrections-Officer-Health-Resources/TWH-Mentoring-Toolkit-Corrections.aspx, accessed on 13 March 2023) [22,23].

Self-selection bias was potentially an even more serious obstacle than cross-contamination, since participants and non-participants were not necessarily comparable at induction. In fact, significant differences were later documented between mentee volunteers and those who elected not to participate.

The main goals of the HMP were to help newly hired COs develop positive health behaviors and to provide them with peer coaching on health and work adjustment topics to prevent the observed early decline in physical fitness, healthy eating, stress management, and work–family balance.

The effort to turn the HMP program over to DOC’s Training Academy (TA) in the last year of HITEC 2 was only partially successful. Following a promising introduction, TA staff reassignments from training to custody, due to a public sector hiring freeze, destabilized the program. However, elements of the mentoring program have continued without guidance or formal evaluation at several facilities up until the present.

***Supervisor Program***. During HITEC 2, in addition to the HMP, the supervisor’s union—CSEA/CSC—launched a parallel design team effort, partially funded through union contract. On request, it was integrated into HITEC 2. Merging supervisors and COs into a single Design Team (DT) had been tried in HITEC 1 at Site B but had not been successful because of perceived CO self-censoring in joint sessions with supervisors present, and also because frequent inter-facility transfers of supervisors vitiated a stable team. The autonomous supervisors’ program was of necessity system-wide rather than facility-based due to limited numbers of supervisors at each facility and the union’s preference for autonomy. The program included a survey, formation of a DT team, training in DT facilitation, and use of the seven-step IDEAS Tool for the participatory design of TWH interventions (https://www.uml.edu/research/cph-new/healthy-work-participatory-program/generate-solutions/, accessed on 13 March 2023). Corrections supervisors used participatory root causes analysis methods assisted by study personnel to amend the standard HITEC study-wide survey for corrections. In HITEC 1, the standard study survey, also called the CPH-NEW All Employee Survey, appeared to be affected by suppressed or muted responses. Self-reported levels of work stress, work–family conflict, and substance misuse were lower than self-reported levels of the same variables in a manufacturing comparison group, although EAP solicitation, disease prevalence, and focus group responses predicted a higher level of dissatisfaction and morbidity. This motivated the generation of a workforce-advised and internally tested set of survey items for CSEA/CSC that were customized for corrections. This participatory design process was replicated study-wide for subsequent surveys, although it did lengthen survey development.

The contractual bargaining for annual training was another novel feature coming from this internally generated DT process. The Supervisors’ DT identified sleep deficiency as the single most addressable problem: 68% had <6 h of sleep per night. Aided by study personnel, the Supervisors’ DT developed a customized sleep app that could be managed on a smartphone. Notably, addressability was not coincident with priority. Extended hours were recognized as the salient risk factor. However, there was sensitivity regarding a loss of time-and-a-half overtime pay, and this was insurmountable at the current time.

In HITEC 2, there was an oversight function adopted by a Study-Wide Steering Committee, consisting of administration represented by labor and leaders of DT and KETs. The group was limited by the absence of senior administrators able to make fiscal decisions, and, more seriously, by irregular attendance by administrators. Not incidentally, this was the same problem encountered by Strickland et al. [24] when they attempted to transplant the HITEC approach to a retail setting.

**HITEC 3** began with modest ambitions: a final 5-year follow-up of the mentored cohort, and a plan to establish labor/management intervention teams at multiple facilities. The plan was to modify existing health and safety, work stress, and quality of work life committees to include a participatory health and well-being component. Given that neither the KET nor the DT had proven to be sufficiently durable to proffer sustainability when the study team presence diminished, we were skeptical that this intervention program would outlast its 5-year federal funding cycle.

Unexpectedly, HITEC 3 took a radical turn. The CT DOC senior leadership committed to participatory teams as a departmental objective, and the bargaining units committed themselves to the ongoing sponsorship of DTs. The initiatives were not entirely de novo since a labor–management Study-Wide Steering Committee (SWSC) had overseen HITEC for a decade. SWSC ebbs and flows had been checkered, depending on varying levels of labor and management engagement. At the start of HITEC 3, the SWSC undertook a period of extensive evaluation, examining its own role in the successes and sustainability limitations of HITEC. An underlying critique was centered on the gap between a shared sensibility from labor and management towards workforce health risk, and a modest level of prioritization. As part of the process, the CT DOC Commissioner engaged in an active participatory role centered on the importance of mental health and well-being. The change was qualitative, unsolicited, and unplanned. HITEC 1 had established the superiority of a DT-led intervention compared to top–down administratively rendered best practices. HITEC 2 had demonstrated efficacy from both a stable DT and task-oriented kaizen teams. However, neither had synthesized a program that would persist with the diminution or eventual disappearance of the academic HITEC research group. HITEC 2 had also demonstrated the acceptability of a CO-led mentoring program, but its high threshold for academic/professional engagement was inconsistent with a modest allocation of internal DT DOC resources.

HITEC 3 had four distinct elements. First, health evaluations were performed on the HITEC 2 cohort of mentees and controls in the HMP to inform a 5-year follow-up window on long-term health effects. Second, after a decade of experience with participatory action, the CT DOC bargaining units enlarged their role and promoted DTs in each geographic region. Third, because the expansion of relatively autonomous sites raised the risk of intervention anarchy and non-comparability from a research perspective, a system of intervention priority ranking was developed to promote the adoption of a common intervention focus for inter-site comparability. Because there were distinct represented constituencies—SWSC administrators, supervisors, line COs—each requiring a specific sampling strategy, congruence methods that used congregated samples, such as DELPHI, were deemed inappropriate. Priority setting began with surveys, followed by clarifying focus groups. Review of these priorities by the SWSC increased organizational readiness to support the formation of DTs and their later intervention proposals, with all parties in agreement to move forward. The methodology used in the priority setting is presented in detail in Table 1 [20], which also offers a mechanism for maintaining input from separate interest groups and homogenizing disparate preferences into common cross-site initiatives. Fourth, project governance was revised, and a small labor–management–academic Small Steering Team (SST) was afforded more extensive decision-making authority on issues of workforce health and well-being. All these efforts were hindered but not derailed by COVID-19, which forced a greater reliance on virtual formats and curtailed staff availability.

HITEC 1 and 2 offered additional lessons that were incorporated into the HITEC 3 reappraisal. The earlier effort to form a combined DT of supervisors and line officers had been unsuccessful at Site B in HITEC 1 but was viable in the HITEC 2 KET program. However, gradual erosion and reliance on external instigation proved problematic. Another lesson was that existing state-mandated committees at DOC, such as the Health and Safety Committee and Quality of Work Life Committee, were dismissed by the existing DTs as inferior to free-standing DTs. While these standing committees simplified scheduling and had a standing membership as well as a joint labor–management axis, they lacked the extended capacity to support TWH-type health and work organizational integrated interventions. Such integrated interventions are characterized by attention to traditional taboo areas like personal mental health, and by directed effort to challenge organizational culture rather than to accept its exigencies.

Perhaps, the most important legacy of HITEC 3 was the resolution of the weaknesses of project oversight due to inconsistent engagement and attendance by senior management and the inability to conform actual representation on the Study-Wide Steering Committee with fluctuating and back-up attendance. At the inception of HITEC 3, the Commissioner of Corrections made a top–down administrative decision to secure senior management involvement and ironically to develop the structure for the full autonomy of the oversight group.

**HITEC 4** is the current extension of the HITEC Program, with financial support from the State of Connecticut replacing federal grant support. During HITEC 3, it had become clear that the long-term prospects and expansion of the HITEC research program were no longer possible due to limited federal resources and a recognition that program emphasis needed to evolve from research to operations. At this juncture, it was unclear whether another external funding source was feasible. Therefore, the final two years of HITEC 3 forced the presumption of loss of research staff support and eventual DT elimination. This forced the consideration of an alternative, entirely internalized and self-sustaining CT DOC program with a different and more consultative level of academic support for training and program evaluation being formulated. DTs were engaged in a series of interviews and surveys to envision these various scenarios. There was, however, a universal preference for maintaining academic involvement to support continuity. Thus, when federal funding did end, DOC and the DTs were already preparing for a next stage.

A key feature of HITEC 4 has been the training of senior managers, specifically executives, directors, wardens, and deputy wardens on effectively working with and supporting grass roots DTs. Training includes familiarization with the goals and operations of an HWPP and practice with applied scenarios. Another feature has been the development of a new governing structure, representing a commitment to long-term program sustainability while de-emphasizing research, and incorporating key intuitional training and communications resources. All workforce-generated interventions were still obligated to achieve feasibility and practicality in order to receive resources controlled by management and administration, both at the local (facility) and systems (departmental) levels.

## 2. Methods

This section serves the purpose of introducing key milestones and evaluative conclusions over a 17-year research project. It also provides background references for associated measurement tools. It does not refer to the methods used specifically in any single HITEC study.

***Site Selection***. Initial site selection was introduced under HITEC 1. Comparable size, security level, staffing, and physical facility were assembled as administrative data. Openness to interventions, “readiness to change”, and cultural homogeneity were determined from a preliminary survey of supervisors. That survey was distributed and collected by the CSEA/CSC Supervisors union.

***Overview of Health Outcomes***. Health outcomes were measured using a survey, focus groups, and interviews; through physiologic function and performance testing; and with a standardized Health Risk Appraisal (HRA). The HRA was dropped due to deficient robustness compared with the core survey. The core survey was a variant of the CPH-NEW All Employee Survey, adapted to corrections. Core surveys were administered at the initiation, mid-point, and conclusion of HITEC 1, and at the initiation and conclusion of HITEC 2. Physiological testing was conducted at each of the four testing intervals at initiation and conclusion, in 2008, 2009, 2011, and 2015. All testing intervals, except for the HITEC 1 mid-point survey, were submitted for longitudinal analysis. The T1 mid-point survey was non-incentivized and was dropped from long-term analyses due to low participation rates.

***Physical Assessment***. The musculoskeletal health physiologic evaluation was based on the Finnish longitudinal study of municipal workers both to provide a basis for international comparison and to assess the validity of the test selection process assumed by the Finnish investigators [25,26,27]. The physiologic test battery included measurements of strength, power, mobility of the spine and trunk, and endurance. Table 3 lists the HITEC tests, their source, and purpose. Inclusion of physiologic tests reflected an inference from Finnish investigators that these appeared to be more predictive than survey data. There was a presumption that results would be age-sensitive and intervention-induced effects would be measurable over an 18–36-month interval.

***HITEC Core Survey***. The HITEC Core Survey was utilized in the longitudinal assessments, and it served as the basis for DT generated surveys and mentoring surveys. The Core Survey was amended and structurally reduced for each iteration. At initiation, it had more than 25 domains. It was based on the “All-Employee Survey” from the CPH-NEW R2P Toolkit (https://www.uml.edu/Research/CPH-NEW/Healthy-Work--priorities/Survey-Manual.aspx, accessed on 13 March 2023). The goal was to select widely used validated instruments, thus enhancing inter-study comparison. This preference for transmissibility was tempered by observations from Schaufeli and Peeters [5], who questioned the use of standard domains in corrections, and the evidence we found of floor and ceiling effects in the response patterns to standardized items that can be attributed to unique demands of the corrections environment. Our corrections variant was, therefore, designed for use by supervisor and CO DTs. Consultative assistance was not, however, modest because item prototyping, formatting, and coding procedures required a greater time commitment than conventional focus group vetting. There were three additional objectives: (1) creation of a reduced item survey capturing most core survey domains; (2) scale reduction through psychometric analysis by removing items not affecting reliability; and (3) selection of symptom items associated with physical examinations.

***HITEC Short Surveys***. Short surveys included pre-intervention assessment tools for individual and DT feedback, and pre- and post- intervention surveys. Short surveys were used in three ways: (1) assessment of effective change; (2) assessment of overall program participation and engagement; and (3) assessment of core health and wellbeing domains likely to be affected by an intervention. The application was to assess health and work environment immediately before and after interventions to mute recall bias. Due to the tendency for corrections personnel to censor responses on standardized survey instruments, HITEC short surveys were revised to include workforce-specific content. A description of several short surveys follows:

i. Intervention Assessment Survey (IAS). The IAS is a short health and environment survey that contains fewer than 10 questions and requires about 5 min to complete. It is intended for DT administration and reflects a reluctance on the part of the population to respond to written or electronic surveys. Domains (general health, physical symptoms, physical and emotional health, work conditions, workplace change, work environment) were factored by the study team from Core Survey responses. The IAS is modular, so the DT can select a specific question set. To allow for pre- and post- comparisons that are identifiable only to the participant, respondents provide their own idiosyncratic code (i.e., phone digits + PIN). The domains and format are IRB-approved so that DTs can choose modules without repeated IRB submission. The form provides de-identified content to the study team for analyses, but it also blinds the DT to the respondent. The format does require that respondents maintain their own unique code to enable follow-up. Follow-up response rates have ranged from <5% to >90%. Factors associated with a high-post intervention response rate include intensity of the intervention, post-survey administration by well-recognized DT representatives, and provision for protected time and space for survey completion. Those contingencies led to a preference for written rather than electronic entry.
ii. The Nutrition and Physical Activity Questionnaire (NPAQ) was developed by the CPH-NEW team. It includes 10 questions adopted from the Hawkes and Nowak nutrition knowledge questionnaire [32] and 26 items that assess eating patterns at the workplace. It was subsequently customized to corrections for weight loss and exercise programs. One reason for customization was specific to corrections where high-calorie ordered-in food, frequent snacking, and bringing in well-packed lunch boxes prevailed due to the need to work double shifts on demand in accordance with their labor contract.
iii. The Food and Physical Activity Liking Survey (FPALS) utilized in this study was customized for direct feedback to DOC users. Participants answer demographic questions, estimate their body size (based on a nine-figure Stunkard Scale [33]), and rate 59 food/beverage items and 10 non-food related items on a general Labeled Magnitude Scale. This was used in the three weight loss programs that were administered by HITEC.

***Qualitative Methods***. Qualitative assessment supplemented surveys. Data were recorded as a single transcript per interview or focus group session and imported into ATLAS TI (version 8, Thomas Muir, Berlin, Germany), a software package designed to handle unstructured qualitative data [34]. Transcribed data were analyzed using the constant comparative method of qualitative data analysis to identify recurrent themes until “theoretical saturation” is achieved; that is, no new themes emerge through subsequent data analysis. In HITEC, all interviews were reviewed in depth by two researchers, and the code structure was reviewed by the full research team for completeness. Independent professional preparation of the transcripts was employed along with IRB review. In addition to standardized coding, an analysis audit trail was constructed to document analytic steps. In HITEC 3 and 4, a grounded theory approach was taken in to identify iterative themes from representatives at different organizational levels, with the goal of streamlining the labor-intensive process used for previous priority settings [35,36,37].

***Exposure Monitoring***. HITEC involved four modes of exposure assessment: (1) survey data, (2) direct observation by the study ergonomist and DT, (3) structured time window analysis, and (4) data logging using electrogoniometric sensors. Data logging proved unsuitable at DOC for security reasons and was replaced by pedometry as a physical activity measure. PATH methodology (Posture, Activity, Tools and Handling) [38] was relied upon for global job assessment. In PATH, observers choose 2 (or more) 15 min “windows” during which they (a) fill out a single, overall work assessment on the cover sheet, including Hand Activity Level (HAL) [39] estimate, and (b) take a visual “snapshot” every 30 s, checking posture and activity categories.

## 3. Results

General findings for different elements of the HITEC studies are referred to in Table 1 and in the text. The following section is structured around participation rates and demographic information. Unpublished longitudinal results will follow in a subsequent publication. These Results do not include previous published data. They do include unpublished results and highlight cross-study inferences.

### 3.1. Participation Rates and Demographic Information

***Longitudinal Studies***. Table 4 presents demographic characteristics from the two study sites.

These are baseline observations from the 2008 inception. Staffing is affected by a relatively early retirement threshold (initially 20 years, changed to 25 years in the last 7 years), frequent transfers (for commuting and shift preference), and promotions. In addition to providing a demographic anchor to better understand results, the comparison affirms that staff characteristics in Sites A and B are more alike than dissimilar. Several characteristics are notable. This is a relatively young work force. There is a high level of reported injury with an annual rate per CO which exceeds one occurrence per year. There are more than 100 job categories in the CT DOC. The largest category of non-CO positions is captains and lieutenants who make up approximately 40% of the non-CO facility-based workforce.

The following table, Table 5, presents combined data collection and measurement dates for Sites A and B. Participation was voluntary, and there were no a priori exclusions. Incentives were offered at all study intervals, except for T2, when there were no incentives and participation levels were minimal. The first survey was the 2007 supervisor’s study. Its purpose was the selection of comparable sites and to appraise organizational readiness. No health information was collected. In general, participation rates were stable across periods except for T2. The decision at T2 to forego incentives was made at union request in the belief that union support would be sufficient. As a result, a combination of USD 50 incentives, raffles, and prizes for test results were offered for subsequent testing with a return to a customary response rate.

The representation of women in the tested workforce at Sites A and B is slightly lower than their overall representation in the CT DOC workforce—27% vs. 30%. There were health differences between men and women as presented in Table 6. Although physical health scores were similar, the male health profile was more adverse, with a greater frequency of hypertension and body fat content, adjusted for gender. Injury claims were similar between men and women. These similarities represent a significant complexity in the ethos of female employees. In CT DOC, there is strong union and administrative support for *Women in Corrections* activities with high levels of participation, but no appearance of selective treatment of women compared with men.

### 3.2. Types of Interventions Resulting from DT Development

Health outcomes are not the only result of participatory interventions. Another important dimension is the intervention process, itself, assessed by participatory engagement, fidelity to the baseline objectives, and coherence of the participatory team. As documented in Figure 2, in HITEC 1, the best practices site (Site A) had limited success: attendance was modest at individual health coaching, weight loss, and chronic disease prevention classes, and participation in a labor–management advisory committee was sparse. Such diffident compliance eliminated the utility of specific health endpoints. At the companion participatory site (Site B), the DT met regularly 1–2 times per month on protected work time and conducted its own surveys and recruitments. Innovative DT programs included improved inter-officer conduct (civility program), the noted footwear emphasis, and DT directed weight loss configured to work schedules. The following items are programs that were developed at both Sites A and B. They reflect differences in the intensity and cost of programs initiated at the two sites.

#### Physical Environment

***Noise Control***. At Site A, a chief complaint was “facility din”, whereby high noise levels, particularly in common areas, overrode officer radio signals. Extensive sound level monitoring was conducted by a university industrial hygienist. Radios were costly but still lacked a customized fit. The labor–management group required extensive involvement by acoustics specialists procured gratis by the study team. A USD 30,000 sound control system was designed for a model pod unit with 130 inmates. It was rejected due to cost. An USD 11,000 acoustical redesign of a common area was also rejected due to cost. A superior and less costly in-the-ear hearing bud was rejected due to complexity, and out of systems cost, since the in-the-ear devices lacked an approved vendor.

***Shoe Insert and Floor Mat Program***. At Site B, knee and hip disorders and falls while attending to emergencies were a major source of reported injury and lost work time. Three issues contributed: smooth floor surfaces (largely a corrections safety feature), officer conditioning and musculoskeletal health, and footwear. Cushioning floor mats were recommended and tested by the DT. A one-month trial of the mats showed improved comfort among 50% (10/20) of participating officers who were normally tasked in the same location for long periods. The cost of the mats was <USD 100 per unit and was accepted as a permanent solution. In addition, cushioning insoles were obtained at a reduced cost from a designated state vendor, but their benefits were not established.

***Indoor Air Quality (IAQ)***. Site A included two adjoined buildings, one being the most antiquated in the DOC system. Studies on air circulation and temperatures in hot weather months were performed. CO concerns were primarily with indoor pathogens. These were ruled out. Facility leadership and DOC administration recognized the IAQ problems as longstanding but demurred because of cost. Ten years after this investigation, the facility was closed.

The IAQ intervention at Site B involved a yearlong effort by the DT. Workforce suspicions of arbitrary temperature changes were disproven through DT recordkeeping. The environmental problems were air-circulation-based, aggravated by inmates blocking registers to prevent cold air entering their cells. The core problem was the age of the system. This was well known to CT DOC, but major changes were deferred due to cost considerations. The DT proposed three plans for ventilation change, beginning with local hygiene including duct cleaning, extending into area duct reconstruction, and in the most complete case, complete system replacement. The program included HVAC training by the State of Connecticut for both COs and inmates, and pre- and post- surveys of COs to assess change. It was a model intervention due to the sophistication of the progressive environmental alterations. The persistence of the DT had the unexpected result of replacement of the main HVAC system. The initiative that involved inmates regularly cleaning HVAC ductwork was so successful that it appeared at other facilities not part of the HITEC Program.

***Weight Loss Program***. The DT sponsored a weight loss program (Site B) which had 82 registrants, 71 participants, and 16 who completed the program. This participation rate was more than twice as high as a HITEC directed program at the matched professional site (Site A). To date, this has been by far the most successful weight loss program undertaken at CT DOC.

**Figure 3:**
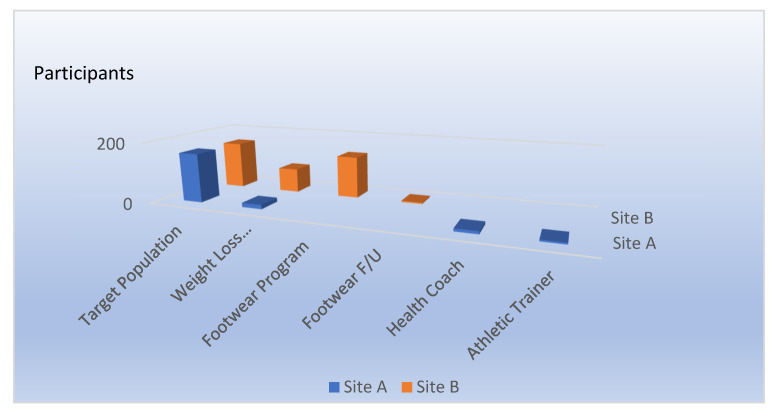
Comparative program engagement at sites A and B.

In addition to general surveys at Sites A and B (T1–T5), there were more specific measures of participation around targeted interventions. Figure 3 presents participation in HITEC 1 for specific interventions. Only the Target Population and the Weight Loss programs are directly comparable due to the different intervention mandates at the two sites. One observation is that the looser DT sponsored weight loss program at Site B had a much higher level of participation. The footwear program was an intervention unique to Site B. There were 138 CO participants, and 41% of the eligible workforce participated and completed lower extremity musculoskeletal surveys. Form-fitted insoles were the non-monetary incentive. Only 18 participants complied with the request to complete a follow-up survey. The Trainer and Health Coach options were unique to Site A and were not offered at the Site B. Despite significant promotion, as already noted, participation was low ~10%. Any conclusions are necessarily inferential, but they confirm the greater level of response to CO lead interventions, despite lower costs and extramural engagement. Perhaps most striking was the success of the supervisor’s sleep app training presented in HITEC 2. Eighty-eight (88) of ninety-two (92) participants completed follow-up in this intervention that was entirely supervisor-run. By comparison, the footwear follow-up survey completion, which was administered by study staff and is presented in Figure 1, is consistent with a far lower level of workforce acceptance.

HITEC relied on different survey formats for specific populations. Different recruitment approaches included incentivization, written and electronic surveys, and direct survey administration by DT personnel. Because COs are not permitted to bring cell phones into custody areas, there are obstacles to online survey administration. Nevertheless, successful interventions did not require incentivization, unlike participation in surveys and physiologic assessments. A more curious observation involves capital costs. Initial requests for capital investment were invariably turned down. The most extensive capital investment was the Site B ventilation system replacement. DOC administrators acknowledged that the persistence of the DT was critical to success. In general, response rates gradually increased throughout the introduction of interventions.

### 3.3. Peer Health Mentoring

Among 406 eligible cadets in the HMP classes, 183 (45%) elected to participate. The participation rate surpassed 50% as the program progressed. Most of the mentees were male (76.4%), a percentage that equaled the proportion of males in this population. Table 7 summarizes the participation of mentors and mentees among the 13 participating facility sites.

The physical health quantitative measures of BMI and body fat percentage increased significantly in the HMP and Control groups from baseline to T3 follow-up. Hypertension increased from baseline to T3 significantly more in the Control group than in the HMP group, but the difference had receded by T3 (see Table 1). Mean depression scores significantly increased in both groups from baseline to T3. Chronic disorder diagnoses increased over time, with more participants reporting doctor-diagnosed chronic disorders at T3 in all groups. At 5 years, no significant differences in health outcomes, health behaviors, and working conditions were observed. However, there was evidence that the quality of the mentor–mentee relationship did affect health outcomes.

## 4. Discussion

HITEC 1 had established workforce preference for DT-led participatory interventions compared to a top–down administratively conceived best practices program. HITEC 2 demonstrated efficacy from both a participatory and task-oriented kaizen team. However, institutionalization was unsuccessful, as neither intervention had synthesized a program that was sufficiently resilient after the complete transfer of control from the academic sponsoring group to an internal DOC group. In comparing the DT and KET approaches, each had strengths and weaknesses. The DT was particularly effective in working through complex initiatives requiring innovation over time but was sometimes slowed down or blocked by the exclusion of supervisors and administrators; the KET was effective in targeted interventions that included supervisors but was overstretched by the complexity of the interventions, and the gradual domination of the KETs by attending supervisors or the deputy warden or the warden. Shorter-term evaluation and feedback, in contrast to less frequently assessed long-term health data, were useful for operations for both groups, particularly since the complexity of large surveys diluted temporal tethering to the actual intervention. The SWSC continued through HITEC 1 and 2, but representation fluctuated between the union-predominant and administration-predominant attendance and motivation, thus mitigating against stable labor–management coordination. The SST successor to the SWSC has been much more effective, thus suggesting an emphasis at inception on committed representative governance.

### 4.1. Participatory Design

Participatory Design Teams require substantial time investment for training, for planning and development of interventions, for development of assessment tools, and for assisting with intervention implementation and iterative design. Short-term health endpoints, such as weight loss or blood pressure control, are elemental in worksite health promotion, but they should not be confused with a horizon of cultural change that is prolonged and should persist through the duration of work tenure. Long-term here means extension into health after retirement when morbidity and mortality accelerate. The progression of HITEC interventions into areas of work organization and mental health has little overlap with term-limited worksite health promotion approaches.

In its baseline articulation, HITEC’s participatory approach was predicated on a paper that highlighted participatory ergonomics as a workable framework for integrating health promotion and occupational safety and health. In its first iteration, HITEC had entertained mirroring successful labor–management cooperative activities, such as health and safety committees that fit into more traditional workplace culture. Mental health and work–family conflict were avoided, given expected resistance to exposing personal information or compromised confidentiality, and they also skirted the mandatory reporting of substance use. HITEC 3 was built on several years of workforce experience with participatory concepts, and the clear interest in aspects of mental health contradicted the expected reticence. The rejection of the best practices approach in HITEC 1 was not the only deviation from conventional expectation. The background literature did not prepare the study team for the limited value of many traditional survey instruments in assessing interventions in corrections, although Schaufeli and Peeters [5] had forewarned the problem of translation to corrections. The effectiveness of surveys informed by workforce DT input produced more variation in responses than traditional “validated” survey items.

An important procedural challenge involved identifying the demarcation between concise DT recommendations that were suitable for administrative takeover and more complex interventions that provoked DT diffidence on total turnover. A related consideration, propelled by COVID-19, was whether short-term situational diversion detracted from the DT ethos of continuous improvement. The experience of DTs shows, so far, that a mature team can tolerate short-term exigent diversions, albeit conditionally. In some cases, such as weight loss, the content is not unique, and it can be replaced by consultant or administrative approaches. Whether they are undertaken by administrative intervention or taken up by a DT, they do not require the sophistication and change in work culture that a DT can bring. DT engagement has the unintended advantage of compliance with the frequency of promotion and transfers in the corrections workforce. The redistribution of personnel experienced with the participatory process between facilities and a high level of promotion of CO facilitators to supervisory ranks forms a type of natural institutional dissemination. Given these contingencies, a continuing responsibility for HITEC is to streamline and shorten continuous improvement process cycles. It is also true that prolonged commitment produces value in the face of resistance or stasis. Put another way, changes in culture and climate in corrections are compatible with the long-view perspective of DTs, even if start-up requires time investment and patience.

### 4.2. HITEC Program Sustainability

In 1987, CT DOC introduced workforce-oriented interventions including allocation of mental health resources, focus groups, and work–family events. The initial positive response was followed by inability to internally sustain the program, and then complete abandonment. CT DOC had also attempted a system-wide wellness initiative, in parallel to HITEC 3. This EWellness program was endorsed by labor and management but petered out despite administrative commitment and engagement of external consultants. Even with solicitation of workforce input, barriers to self-perpetuation that arise in top–down interventions inhibited self-perpetuation. Participatory design at the grass roots level is not, however, a simple panacea. CT DOC had introduced its Employee Assistance Unit (EAU) which is based around individual interventions and is managed by trained COs and supervisors. This is a companion activity which reflects an organizational commitment to staff health and well-being. However, the necessary reliance on trained professionals had produced disillusion in line officers due to the almost inevitable dilution of direct influence. The successes of HITEC rest on a broader but less professionalized peer representation and control on a continuous basis. If there are lessons, one is that overlapping initiatives are not necessarily distracting, and different approaches to health and well-being can co-exist. Another is that well-led and well-intended administrative programs have an essential risk of loss of worker engagement over time.

The HITEC experience with PAR, participatory intervention design, and participatory program design merits some comment on sustainability. For HITEC, there has been a consistent transfer of responsibility to CT DOC staff. However, it remains too early to tell whether this process can or even should exclude all input from focused professionals going forward, either from the original academic sponsor or another quasi-independent group with a suitable skill base.

### 4.3. Workforce Centricity and Labor Representation

To characterize their work, one of the DTs had provided the following epithet:

“By officers, For officers”.

Workforce-centricity has not required that the academic or professional sponsorship should entirely disappear. As DT sophistication deepens, there has been continued interest in more sensitive surveys and evaluation instruments, and in derivative programs such as cardio-vascular disease analysis and sleep analysis.

The role of bargaining units and union leadership was central. HITEC began with a presumption that senior management endorsement was highly probable, given earlier management efforts to advance workplace health promotion. But contrarily, a nominal “buy-in” from labor was insufficient for program success. Commitment and responsibility from labor and management in a somewhat co-equal arrangement was the pre-requisite for work culture change. Thus, the first HITEC efforts were invested in securing union engagement prior to an approach to management. A discrete union-based program was not a promising prerogative; however, because without the provision of resource support from management, particularly protected time and scheduling, intervention planning and subsequent interventions were destined to fail. Furthermore, initiatives from the bargaining units alone posed a barrier to organizational change.

In HITEC 3 and 4, more than a decade of PAR experiments and participatory interventions appeared to have flourished from established vigorous roots. HWPP intervention planning required a DT to address resource needs and obstacles as well as budgets and detailed implementation plans for the practical transfer of responsibilities to management. This intervention planning process requires DT sophistication which was aided through the use of the IDEAS Tool designed to support participatory intervention planning and developing a business case for interventions that could be presented to management [20]. Nonetheless, CO-directed bargaining units often lacked the administrative culture which is stronger in supervisors’ units. Therefore, ambitious DT plans by COs in some cases risked exceeding the skills of DT members. At onset, it was yet unclear whether interventions would be turned over fully to management as a finished package, whether the DT could and should continue in some fashion into intervention implementation to assist with an iterative design of the intervention, or whether it would dissolve after handing the intervention off to management and surrender to a new DT and intervention focus. It became clear in HITEC 3 that DTs were capable of replenishing themselves and moving on to new projects. It was also clear that most DT members preferred to remain in a key role with implementation, motivated by apprehension that full withdrawal from project execution would dilute fidelity to the grass roots principles underlying complex implementations. Simpler discrete interventions, such as computer purchases were amenable to simple hand-off.

The extended duration of the HITEC Program also provided a platform for the training of line officers, many of whom would later progress to supervisory and management positions after displaying leadership in HITEC. After 16 years, despite the many changes in personnel, there was prescriptive understanding of the participatory process, and programs emanating directly from the workforce had far greater likelihood of acceptance and effectiveness than administratively sponsored programs, thus reflecting an important cultural change in support of *Total Worker Health*.

Shared aims by labor and management are essential for cultural change, but they do not eradicate differences in operational experience and ability to harbor resources. In a related vein, workforce involvement overlaps but is not fully congruent with bargaining unit priorities. Participatory action demands subtlety and flexibility that is not normally addressed by the adjudicative mechanisms of the collective bargaining process and its defined cycles. There are also strong workforce health champions who are relatively indifferent to union affairs. The experience from HITEC 2 corroborated the importance of ongoing bargaining unit leadership participation for successful interventions [9]. This is likely to be even more important in an institutionalized future for the HITEC Program since there is an underlying tension between the incorporation of labor involvement and control over new dedicated positions. In some cases, DTs might appear to compete with existing labor–management teams, such as health and safety committees, or as a rival to union representation. The distributions of influence are subtle. For example, in HITEC, the supervisor’s unit introduced participatory health and wellness activity into their contract. On the other hand, the COs executive board representatives initially rejected the labor–management vetting of mentors as an intrusion into bargaining units’ prerogatives, and some have resisted incorporating HITEC-type activities into their contracts. HITEC was able to avert the concerns over bargaining unit vitiation through extensive compromise and discussion, thus evading conflicts that undermined, for example, the United Auto Workers GM joint work and health initiatives in the 1980s [40]. In HITEC, the most skilled labor leaders understood the necessity of a diverse DT and the importance of participants who were dedicated to workforce well-being but were not obligatory participants in bargaining unit affairs. However, all these intricacies do not disguise the fact that 16 years is a very long time for academic researcher engagement with a field population, and that the extent of representation, let alone the quality of union leadership, is variable. Moreover, the HITEC experience occurred in a represented labor environment, and the absence of some administratively skilled labor representation imposes a significant but manageable barrier to program success.

### 4.4. Importance of Established Governing Structures and Resiliency

Key personnel are impermanent in corrections. The engagement of the key institutions in corrections—union representatives, supervisors, senior administrators, wardens and deputy wardens, and internal training assets—also means that failure or stalemate in one arm does not jeopardize the entire process. Redundancy and the avoidance of reliance on a single institutional resource was key to resilience. Changes in union leadership and variable attitudes towards cooperation with management are barriers, but also work against granitic stalemate. An appreciation of the formative role of the DT may be a central realization. While the tendency is to consider intervention results above all, the DT can become a core institutional asset, and a well-constructed DT training has proven to be energizing.

Changes in administrative and union leadership have introduced alternate patterns of resistance, rejection, and endorsement. The HITEC experience has been that diffidence erodes. As long as representative groups are operative, delay rather than fatal unwinding has been the outcome to date. Also, longstanding health issues to not magically disappear. The successes of HITEC 3 suggest that a pattern of dormancy and compressed action may be the norm in corrections. These experiences with temporal factors may help abbreviate replication in other corrections environments and offer encouragement to continue through downward cycles. The development of a stable oversight structure that is trained to enable participatory action does generate a curious contradiction. Accepted principles of participatory work dilute reliance on top–down mandates and policy requirements. However, without inscribed policy changes, successful programs are subject to dismantling or decay, particularly in a hierarchical organization like corrections. Sixteen years may be insufficient time to fully characterize this type of multi-directional contradiction. Perhaps the most important single lesson from HITEC has been the centrality of the DT, maintained through an ongoing revising and retraining in its structured approach. DTs have recognized the centrality of periodic return to formative basics and fundamental health issues when direction and motivation seem to stall or dissipate. Fidelity to collective problem solving has proven to be an enduring asset. Skilled senior management is faced with maneuvering between the facilitation of workforce autonomy and institutionalizing change through policy making. It was only in HITEC 4 that the importance of management training for working effectively with front-line worker led DTs was translated into management-specific training efforts and materials and the recognition of the need to repeat these trainings on a regular basis to address management turnover. Workforce participatory programs require more than intermittent inclusion on managerial agendas. Perhaps one of the most important lessons was the primacy of executive upper middle-management training. While time constraints are an eternal barrier, managerial training was the one identifiable step that abbreviated the length of induction of new DTs for program expansion. In an organization, like corrections, where promotions, retirements, and transfers are frequent, the iterative training of senior managers plays a necessary role. The usual resistance comes out of time shortness and presumptions of knowledge. It is a fundamental test of organizational commitment to establish managerial involvement as a priority. Members of the HITEC team have worked in settings outside of corrections, and we have concluded that disciplined commitment from senior managers is an acid test for organizational readiness.

### 4.5. Comparing Community and Workplace Interventions

There are fundamental ways in which a worker population organized around dedicated health and workplace design processes is distinguished from a group organizing a community intervention. For example, information gathered through interview and narrative retrieval by the study analyst in CBPR [41] was approached quite differently. The multiple steps of the IDEAS^®^ begins inductively with a root causes analysis approach and proceeds reductively to a specific intervention(s) which is explored pro-actively for cost, expected efficacy, practicality, and resource requirements. The short surveys presented in the Section 2 offer an efficient process for evaluating effects. This is, of course, possible when the DT can meet and harbor resources, at least partially within the work day. Workplace-based interventions also reduce the multiplicity of associative factors that are inherent to community-based interventions, such as interacting components and the tailoring of interventions [42].

Some investigators have raised the familiar concern about the overall utility of CBPR due to excessive input in contrast to the neglect of key considerations [43]. Another concern has been the problems raised around the protection of human subjects since the individualized consent process features protections that largely exclude community autonomy and practical resource limitations [44]. There are different considerations which arise in both observational surveys and interventions on the working population. There is particular concern about identity protection revealing information that may affect employment. In HITEC, worker representation played a core role in the consent process, as a worker’s committee was asked to vet all surveys and tests. The results have been more protective than usual IRB requirements, which again reflects the differences between research performed in the workplace and the broader community. In HITEC, a strong union presence contributed to study acceptance by the larger workforce than a barrier to participation.

Finally, we return to the most important factor that differentiates participatory workplace research from community-based research. It is the governing or oversight structure. Unlike CBPR, the identification of democratic representation or overlooking of underserved populations are not the most serious barriers. In the workplace setting, the hierarchy of authority, both fiscal and administrative, is either a main barrier or a main facilitator of an intervention. As discussed above, this is most addressed in the description of HITEC 4, where the iterative training of senior managers (commissioners and deputies), next-tier senior staff (directors and division directors), and facility-based leadership (wardens and deputies) has been approached directly with training on the participatory DTs and their roles as facilitators.

### 4.6. Mentoring

Our conclusion on the mentoring program was definitive. The program piqued a high level of CO engagement. However, the professional effort required to maintain an intense mentoring program for all new cadets was not justified given the extent of the effort and the overwhelming effects of employment in corrections, which superseded individual mentoring for a one-year period. We did not secure a prolonged treatment effect despite a positive review of the program by participants at the end of the one-year mentoring sequence and mentees volunteering to become future mentors. Instead, we concluded that an individual health mentoring program was not sufficiently effective in the absence of synchronous change in work culture and work organization. Consistent with a TWH model, the adverse systemic effects of working in corrections were too potent to be reversed individually. We also concluded that mentoring impacts would not persist unless the availability of peer counseling or an equivalent for onsite mental and physical health was also ongoing. These lessons have been incorporated into HITEC 4, where the conclusions regarding next steps are far from bleak. Some elements of the HMP remained intact in several facilities, and mentoring materials were reused and inserted into the FTO program. In a corrections environment where an ongoing culture of change is rooted, the margins of success and failure may simply exceed an a priori timetable.

### 4.7. Multi-Level Organizational Strategies

Over the 16 years of HITEC, there have been six CT DOC commissioners. At the two most studied sites, there had been five changes in wardens over a decade. There have been changes in union leadership in each of the four participating locals, with varying levels of engagement by new leadership and different attitudes towards labor–management cooperation. The need is for workforce-based intervention programs to be multi-level and robust so that reverses in one component do not undo essential processes. Constant transition is typical of the corrections sector. Self-sustaining organizational structures require a solution of personnel changes so that reform initiatives are not undone. In HITEC 3, processes were initiated to maintain continuity in key union and management leadership functions.

## 5. Conclusions and Remarkable Findings

The following is a summary of important insights that were not necessarily anticipated at the onset of HITEC 3 and HITEC 4.

- DTs proved to be more resilient and capable of regeneration than expected. The HWPP presupposed that DTs would devolve and require assistance from the SWSC and facility leadership to persist. The DTs proved highly successful at internal replacement and longevity.
- DTs were not satisfied with simply handing over their intervention plans. In all cases, they were interested in carrying on through the implementation phase: for example, assisting with the evaluation efforts of intervention processes and outcomes.
- Cooperative work and engagement produce synthetic solutions. Perceived a priori barriers, such as the inertness of facility-based teams and the supervisor versus CO division, were difficult to resolve in a priori planning but were successfully addressed in practice.
- Cultural change is a long-term continuous process. The importance of supporting an ongoing effort was intuitively grasped by DTs and the workforce.
- The centrality of key issue identification and gauging sizes of effects through surveys was implicitly understood and was not seen as an academic imposition. The acceptance of quantifiable outcomes was likely a product of both curiosity and lengthy experience with the academic team, that had established trust.

TWH presupposes that the creation of a long-term and sustainable workplace health culture requires the integration of conventional health and safety with individual well-being. NIOSH, CPH-NEW, and CT DOC administrators and front-line workers made continuous investment through three rounds of federal funding. As can be expected, the outcomes are still evolving within what has become an innovative system-wide initiative to *promote safe and healthy work design and well-being.* There are three obvious implications that underwrite the current character of HITEC. First, interventions having this degree of sophistication, and sector-wide application reflect broad comprehension. A multi-level commitment of research to practice should not be left entirely to dangle from past accomplishment once certain research objectives are realized. Second, the scientific understanding of the principles necessary for sustaining a successful transformation of organizational culture is undeveloped, and research-informed observation can enhance efforts for continuous improvement. Third, the prolonged induction time invested in research objectives was, in part, the result of special circumstances, such as a skilled professional team, and ongoing investment that lead to state support. However, successful exemplars have the potential to simplify transfer to other contexts. These can serve as a basis for scaling up and as a basis for disseminating the most effective programmatic approaches elsewhere in the same sector. In this respect, and despite COVID-19, the existing DTs were unanimous in their commitment to translating their work into expansion and dissemination. In fact, the DTs were demonstrably encouraged by a laudatory assessment from the Surgeon General and the acknowledgment of their efforts by Scandinavian colleagues. One lesson is that while procedural shortcuts may curtail induction time, significant organizational change, as we have seen in CT DOC, is a long-term investment that is characterized by rewards that are larger than unexpected.

## Figures and Tables

**Table 1:** Prior publications from the HITEC team relevant to this article.

Topic Area	Citation	Findings and Key Themes
**Participatory Approaches Utilized in HITEC**	Henning, R.; Warren, N.; Robertson, M.; Faghri, P.; Cherniack, M.; CPH-NEW Research Team. Workplace health protection and promotion through participatory ergonomics: An integrated approach. *Public Health Rep.* **2009**, *124*, 26–35.	Integrating participatory ergonomics with traditional health promotion approaches in a bottom–up participatory model for engaging employees in innovative iterative design of workplace interventions to benefit worker wellbeing.
	Punnett, L.; Warren, N.; Henning, R.; Nobrega, S.; Cherniack, M.; CPH-NEW Research Team. Participatory ergonomics as a model for integrated programs to prevent chronic disease. *J. Occup. Environ. Med.* **2013**, *55*, S19–S24.	Use of participatory methods for achieving successful workplace health promotion (WHP) programming, and, specifically, the relevance of participatory ergonomics (PE) for the NIOSH TWH initiative.
	Cherniack, M.; Dussetschleger, J.; Dugan, A.; Farr, D.; Namazi, S.; El Ghaziri, M.; Henning, R. Participatory action research in corrections: The HITEC 2 program. *Appl. Ergon.* **2016**, *53*, 169–180.	Programmatic overview of participatory interventions for corrections staff. Description of a peer health mentoring program which had positive health effects at 1 year and in diluting effects from overtime work.
	Dugan, A.G.; Farr, D.A.; Namazi, S.; Henning, R.; Wallace, K.N.; El Ghaziri, M.; Punnett, L.; Dussetschleger, J.L.; Cherniack, M.G. Process evaluation of two participatory approaches: Implementing Total Worker Health^®^ interventions in a correctional workforce. *Am J. Ind. Med.* **2016**, *59*, 897–918.	HITEC-2 comparison of two participatory programs: a CO-only Design Team (DT) with a task-specific Kaizen Event Team (KET). A Program Evaluation Rating Sheet (PERS) was developed and utilized to document and evaluate program implementation. Key features such as level of bargaining unit involvement were identified.
	Cherniack, M.G.; Punnett, L. A participatory framework for integrated interventions. In *Total Worker Health*; Hudson, H.L., Nigam, J.A.S., Sauter, S.L., Chosewood, L.C., Schill, A.L., Howard, J., Eds.; American Psychological Association: Washington, DC, USA, 2019, pp. 107–124.	Participatory action research principles, participatory ergonomics, and other workplace participatory processes are detailed as foundations for an approach to preventing chronic health conditions including heart disease and diabetes. Compatibility with NIOSH Total Worker Health (TWH) is provided as case examples.
**Peer Mentoring and Training**	Namazi, S.; Kotejoshyer, R.; Farr, D.; Henning, R.A.; Tubbs, D.C; Dugan, A.G.; El Ghaziri, M.; Cherniack, M. Development and implementation of a Total Worker Health^®^ mentoring program in a correctional workforce. *Int. J. Environ. Res. Public Health* **2021**, *18*, 8712.	A Health Mentoring Program (HMP) paired newly hired COs with tenured and trained COs to provide health coaching. Goals were prevention of an observed early decline in physical fitness and promotion of healthy eating, stress management, and work–family balance. Methods for training, selection, quality maintenance, and documentation of status by survey and performance testing are described.
	Kotejoshyer, R.; Gilmer, D.O.; Namazi, S.; Farr, D.; Henning, R.A.; Cherniack, M. Impact of a Total Worker Health^®^ mentoring program in a correctional workforce. *Int. J. Environ. Res. Public Health* **2021**, *18*, 8436.	At 5 years, positive health effects in 269 corrections cadets, mentored for 1 year, were no longer observable compared to controls. The authors concluded that the results did not justify the extensive outside professional effort. Successful program elements continue to be adopted.
**Design Team Sponsored Interventions**	Namazi, S.; Dugan, A.G.; Cavallari, J.M.; Rinker, R.D.; Preston, J.C.; Steele, V.L.; El Ghaziri, M.; Cherniack, M.G. Participatory design of a sleep intervention with correctional supervisors using a root causes approach. *Am. J. Ind. Med.* **2023**, *66*, 167–177.	The IDEAS tool, a structured seven-step planning process, was used to develop, implement, and evaluate sleep interventions. Customized surveys and an app were developed by the supervisors’ DT. Reduction of distractions and rumination were effective. Proposed scheduled changes were submitted to administration.
	Dugan, A.G.; Namazi, S.; Cavallari, J.M.; Rinker, R.D.; Preston, J.C.; Steele, V.L.; Cherniack, M.G. Participatory survey design of a workforce health needs assessment for correctional supervisors. *Am. J. Ind. Med.* **2021**, *64*, 414–430.	Corrections supervisors developed their own survey to evaluate health and well-being. There were distinct advantages over conventional survey items, particularly for personal issues of substance use and mental health.
	Dugan, A.G.; Namazi, S.; Cavallari, J.M.; El Ghaziri, M.; Rinker, R.D.; Preston, J.C.; Cherniack, M.G. Participatory assessment and selection of workforce health intervention priorities for correctional supervisors. *J. Occup. Environ. Med.* **2022**, *64*, 578–592.	Methods for survey development compatible with investigator rigor and PAR and CBPR principles are described as developed in HITEC. The use of focus groups and a DT to set health priorities are explained.
**Methods Customized for Participatory Research in Corrections**	Cherniack, M.; Berger, S.; Namazi, S.; Henning, R.; Punnett, L.; CPH-NEW Research Team. A Participatory action research approach to mental health interventions among corrections officers: Standardizing priorities and maintaining design autonomy. *Occup. Health Sci.* **2019**, *3*, 387–407.	Novel methodology for combining multiple sites where principles of autonomy across sites and inter-site comparability are objectives. The direct application of the multi-state priority selection process is presented. Comparison with other methods such as DELPHI is provided.
	Punnett, L.; Cavallari, J.M.; Henning, R.A.; Nobrega, S.; Dugan, A.G.; Cherniack, M.G. Defining ‘integration’ for Total Worker Health^®^: A new proposal. *Ann. Work. Expo. Health* **2020**, *64*, 223–235.	A theoretical approach to homogenizing workplace health with work organizational change is presented. Methods for accommodating conflicting managerial priorities for efficiency alongside Total Worker Health worker-centricity are described.
	Hughes, J.M.; Henning, R.A.; Robertson, M.M. Organizational sensemaking systems as a determinant of successful organizational change: A grounded theory approach. *Proc. Hum. Factors Ergon.* **2022**, *66*, 90–94.	Organizational sense-making is offered as an alternative option to large-scale survey assessments to recognize explanatory points of consensus and priority in corrections cohorts.
**Physical and General Health Issues in the HITEC Corrections Cohort**	Warren, N.; Dussetschleger, J.; Punnett, L.; Cherniack, M.G. Musculoskeletal disorder symptoms in correction officers: Why do they increase rapidly with job tenure? *Hum. Factors* **2015**, *57*, 262–275.	The rapid onset of musculoskeletal disorders in corrections workers compared with manufacturing personnel appears to be a combination of psychosocial as well as biomechanical factors.
	Morse, T.; Dussetschleger, J.; Warren, N.; Cherniack, M. Talking about health: Correction employees’ assessments of obstacles to healthy living. *J. Occup. Environ. Med.* **2011**, *53*, 1037–1045.	Hypertension was a double national norm. Officers related their elevated stress to concerns with security, administrative requirements, and work/family imbalance. Authors concluded that corrections workers are at high risk of chronic disease, and environmental changes are needed to reduce risk factors.
	Mignano, C; Faghri, P.D.; Huedo-Medina, T.; Cherniack, M.C. Psychological health, behavior, and bodyweight (PBBW) model: An evaluation of predictors of health behaviors and body mass index (BMI). *J. Workplace Behav. Health.* **2016**, *31*, 37–56.	Psychological health and reported stress were associated with body weight in corrections officers. Healthy behaviors mediated the negative effect of work stress on body weight.
	Obidoa, C.; Reeves, D.; Warren, N.; Reisine, S.; Cherniack, M. Depression and work family conflict among corrections officers. *J. Occup. Environ. Med.* **2011**, *53*, 1294–1301.	Work family conflict in corrections workers was elevated and associated with high levels of clinical depression. However, responses were muted below expectations, raising questions about the utility of validated surveys developed in other populations.
	Buden, J.C.; Dugan, A.G.; Faghri, P.D.; Huedo-Medina, T.B.; Namazi, S.; Cherniack, M.G. Associations among work and family health climate, health behaviors, work schedule, and body weight. *J. Occup. Environ. Med.* **2017**, *59*, 588–599.	Over 85% of the sample was overweight/obese. Both favorable workplace climate and family climate were associated with a lower BMI. Overtime shift work appeared to share a relationship with higher BMI.
	Buden, J.C.; Dugan, A.G.; Namazi, S.; Huedo-Medina, T.B.; Cherniack, M.G.; Faghri, P.D. Work characteristics as predictors of correctional supervisors’ health outcomes. *J. Occup. Environ. Med.* **2016**, *58*, e325–e334.	Corrections supervisors had a higher prevalence of obesity and comorbidities than the general US adult population. Burnout was significantly associated with nutrition, physical activity, sleep duration, sleep quality, diabetes, and anxiety/depression. Job meaning, job satisfaction, and workplace social support may predict health behaviors.
**Mental Health Issues in the HITEC Corrections Cohort**	Gilmer, D.O.; Magley, V.J.; Dugan, A.G.; Namazi, S.; Cherniack, M.G. Relative importance of incivility and loneliness in occupational health outcomes. *Occup. Health Sci.* **2023**, *7*, 1–25.	General loneliness in corrections officers appears to be an important explanatory factor in emotional exhaustion, job satisfaction, and depression when work stress is controlled.
	Namazi, S.; Dugan, A.G.; Fortinsky, R.H.; El Ghaziri, M.; Barnes-Farrell, J.L.; Noel, J.; Cavallari, J.M.; Shaw, W.S.; Cole, W.A.; Cherniack, M.G. Traumatic incidents at work, work-to-family conflict, and depressive symptoms among correctional supervisors: The moderating role of social support. *Occup. Health Sci.* **2021**, *5*, 493–517.	One-hundred and fifty-six (156) corrections supervisors were studied to assess the effects of direct and indirect trauma on work-to-family conflict and depressive symptoms. Work-to-family conflict mediated the association between traumatic incidents and depression. Social support moderated the association between traumatic incidents and depression. Interventions directed to supervisor mental health and family health are discussed.
	Namazi, S.; Dugan, A.G.; Fortinsky, R.H.; Barnes-Farrell, J.; Coman, E.; El Ghaziri, M.; Cherniack, M.G. Examining a comprehensive model of work and family demands, work-family conflict, and depressive symptoms in a sample of correctional supervisors. *J. Occup. Environ. Med.* **2019**, *61*, 818–828.	In corrections supervisors, overtime significantly predicted work-to-family conflict, and work-to-family conflict significantly predicted greater depression. Overtime work had an indirect effect on depression.

**Table 2:** Comparison of Design Team (DT) and Kaizen Effectiveness Teams (KETs).

	KET	DT
Number of Interventions	4 interventions, each with a separate KET, and each lasting 3 months	4 interventions with a continuous DT and the sole restriction being completion by study end
Intervention Themes	Same as DT	Same as KET
Duration of Each Intervention	Fixed Time Limit/Intervention	Variable Time Limit/Intervention
Participatory Team Composition	Line Staff and Supervisors	Line Staff only
Team Solicitation	Open Recruitment	Labor and Management
Evaluation Tools	Group Surveys and Tests Intervention specific pre/post	Group Surveys and Tests Intervention specific pre/post

**Table 3:** Physiologic testing.

Name of Test	Purpose	Status for f/u
Anthropometric assessment	Height (cm), weight (kg), waist circumference (CM)	Maintained
Body composition/BIA [28]	Body fat	Maintained
Grip strength [29]	Hand strength	Maintained
Max. power/ergometry exercise test [30]	Lean muscle function and v02 max. approximation	Maintained
Functional reach test [31]	Upper body mobility	Dropped due to unreliability
Spine mobility [27]	Spinal intervals	Dropped due to unreliability
Blood pressure	Hypertension assessment	Maintained

**Table 4:** Workforce characteristics at two DOC facilities participating in the HITEC T1 study—2008.

Sites	Total Workers	COs	Participant	Inmate Ratio: CO	Avg. Age Years COs	Age Range	Ann. Illness/ Injury	Illness and Injury Rate per CO	Ann. MS Strain	MSI Rate per CO
Professional (Site A)	434	349	161	4.83	40.6	22–61	625	1.44	210	0.48
Participatory (Site B)	428	340	157	4.39	40.9	21–68	707	1.65	165	0.39

**Table 5:** Response characteristics for five assessments ^#^.

	2007 ^1,2^	2008 ^3,4^	2009 ^2,4^	2011 ^3,4^	2013 ^3,4^	2016 ^3,4^
		T1	T2	T3	T4	T5
Target population	598	862	820	826	841	845
Participants	124	332	65	382	348	390
% Participation	20.7%	38.5%	8.0%	46.2%	41.4%	46.2%
	Supervisors	Sites A and B	Sites A and B	Sites A and B	Sites A and B	Sites A and B

^1^ Supervisors study; ^2^ No incentives; ^3^ USD 50 for survey and physiologic evaluation; ^4^ Core survey, physiologic testing; ^#^ Differences from Table 3 are secondary to completeness of testing.

**Table 6:** Male and female staff health at two DOC facilities participating in the HITEC study—2008.

All DOC Subjects			
**Variables**	**Males**	**Females**	**Total**
Participants	242	89	332
Age: mean (sd)/median	41.3 (8.3)	43.9 (8.6)	42.0 (8.4)
SF-12 PCS: mean (sd)	51.3 (6.5)	51.3 (8.4)	51.3 (7.1)
SF-12 MCS: mean (sd)	48.9 (10.2)	48.4 (10.4)	48.8 (10.2)
**BMI**			
Mean (sd)	32.25 (5.31)	28.72 (5.32)	31.28 (5.53)
% Normal	4.0%	24.1%	9.6%
% Overweight	32.2%	44.4%	35.5%
% Obese	63.6%	31.5%	54.8%
**Body Fat**			
%Healthy	17.6%	55.3%	27.3%
% Overweight	33.1%	21.3%	30.1%
% Obese	48.5%	23.4%	42.1%
**Hypertension**			
% Normal	16.1%	42.6%	23.5%
% Pre HTN	54.5%	44.4%	51.8%
% HTN	29.4%	13.0%	24.9%

**Table 7:** Mentee and mentor participants.

Location	Mentees		Mentors	
	Number	Percent	Number	Percent
**A**	30	16.4	19	18.1
**B**	17	9.3	10	9.5
**C**	14	7.6	7	6.7
**D**	23	12.5	14	13.3
**E**	19	10.4	12	11.4
**F**	18	9.8	12	11.4
**G**	7	3.8	2	1.9
**H**	24	13.1	10	9.5
**I**	20	10.9	8	7.6
**All others (n = 4)**	11	6.0	11	10.5
**Total**	183		105	

## Data Availability

The data presented in this manuscript are available on request from the corresponding author. This was a historical review, not a data generating study. The UConn Health Library can provide research materials from its UCL Data Archive for public access, upon request https://lib.uconn.edu/health/find/databases/ (accessed on 13 March 2023).
